# SOCS1 function in BCR-ABL mediated myeloproliferative disease is dependent on the cytokine environment

**DOI:** 10.1371/journal.pone.0180401

**Published:** 2017-07-28

**Authors:** Özlem Demirel, Olivier Balló, Pavankumar N. G. Reddy, Olesya Vakhrusheva, Jing Zhang, Astrid Eichler, Ramona Fernandes, Susanne Badura, Hubert Serve, Christian Brandts

**Affiliations:** 1 Department of Medicine, Hematology/Oncology, Goethe University, Frankfurt, Germany; 2 German Cancer Consortium (DKTK), Heidelberg, Germany; 3 German Cancer Research Center (DKFZ), Heidelberg, Germany; 4 Hematology/Oncology, Children’s Hospital Boston, Harvard Medical School, Boston, United States of America; Universita degli Studi di Firenze, ITALY

## Abstract

Treatment with tyrosine kinase inhibitors is the standard of care for Philadelphia chromosome positive leukemias. However the eradication of leukemia initiating cells remains a challenge. Circumstantial evidence suggests that the cytokine microenvironment may play a role in BCR-ABL mediated leukemogenesis and in imatinib resistance. Gene expression analyses of BCR-ABL positive ALL long-term cultured cells revealed strong reduction of SOCS mRNA expression after imatinib treatment, thereby demonstrating a strong inhibition of cytokine signaling. In this study we employed SOCS1—a strong inhibitor of cytokine signaling—as a tool to terminate external cytokine signals in BCR-ABL transformed cells *in vitro* and *in vivo*. In colony formation assays with primary bone marrow cells, expression of SOCS1 decreased colony numbers under pro-proliferative cytokines, while it conferred growth resistance to anti-proliferative cytokines. Importantly, co-expression of SOCS1 with BCR-ABL led to the development of a MPD phenotype with a prolonged disease latency compared to BCR-ABL alone in a murine bone marrow transplantation model. Interestingly, SOCS1 co-expression protected 20% of mice from MPD development. In summary, we conclude that under pro-proliferative cytokine stimulation at the onset of myeloproliferative diseases SOCS1 acts as a tumor suppressor, while under anti-proliferative conditions it exerts oncogenic function. Therefore SOCS1 can promote opposing functions depending on the cytokine environment.

## Introduction

The reciprocal translocation of chromosomes 9 and 22 [t(9;22)(q34;q11)] generates the so called Philadelphia chromosome harboring the fusion gene BCR-ABL.[1,2] The non-receptor tyrosine kinase c-ABL regulates a variety of cellular processes including cell growth and survival.[3,4] Fusion of BCR upstream to the ABL gene results in a constitutively active tyrosine kinase conferring proliferative advantages and resistance to apoptosis on affected cells.[5] Depending on the breakpoint in the BCR gene, fusion proteins of different sizes are generated, most commonly proteins of 210 kDa (BCR-ABLp210) or 185 kDa (BCR-ABLp185).[6] BCR-ABLp210 is present in about 90% of chronic myeloid leukemia (CML) patients and approximately one third of B-cell acute lymphoid leukemia (B-ALL) patients. On the other hand BCR-ABLp185 is detected in B-ALL leukemia and is rarely found in CML patients.[7] In murine models, over-expression of the respective protein in hematopoietic stem and progenitor cells gives rise to an aggressive myeloproliferative disease (MPD) or leukemia phenotype with very short latency.[8,9] Although development of tyrosine kinase inhibitors (TKI) was a remarkable milestone for the therapy of BCR-ABL positive leukemias, disease persistence and resistance to TKIs are remaining issues. Mutations in the kinase domain[10,11], elevated BCR-ABL levels[12] and increased STAT5 expression[13] are among the reasons causing resistance to TKIs. Recent studies suggest that CML stem cells are not addicted to BCR-ABL and are therefore not eliminated by imatinib. Cytokine support is a crucial factor allowing growth and survival of CML stem cells independent of BCR-ABL activity.[14] Thus, the cytokine microenvironment is an important determinant in BCR-ABL positive leukemia.

In normal hematopoiesis, cytokines induce diverse cellular responses including differentiation, proliferation or growth arrest. Since cytokine receptors lack intrinsic kinase activity, they require JAK kinase activity to activate the JAK-STAT signaling pathway.[15,16] STAT target genes include inhibitors of cytokine signaling to ensure accurately timed signal termination. The induced suppressors of cytokine signaling (SOCS) family members (CIS and SOCS1-7) inhibit the cytokine signal in a classical negative feedback loop employing several complementary mechanisms.[17–19]

Diverse and some contradicting roles were ascribed to SOCS proteins in cancer. SOCS1 was the first member of this protein family to be discovered.[20,21] Together with SOCS3 it is the most analyzed SOCS protein. A large body of literature deals with the investigation of SOCS1 and SOCS3 in malignant diseases especially in the hematopoietic system demonstrating the importance of SOCS1 and SOCS3 in hematological disorders. In various cancer types decreased SOCS1 expression was detected indicating a tumor suppressive nature.[22] Epigenetic inactivation of SOCS1 by methylation of the promoter or mutations of the SOCS1 gene was frequently observed.[23–25] In contrast, in breast cancer tissue SOCS1 is higher expressed than in control samples.[26] Similarly, in melanoma cells higher SOCS1 expression levels than in the corresponding normal tissues are detected.[27] These observed discrepancies in different cancer types require a more comprehensive investigation in a context specific manner. Interestingly, in primary material from CML patients high levels of SOCS2 were detected. But, despite elevated SOCS2 expression in patients, SOCS2 was found to be dispensable for BCR-ABL induced CML and for normal hematopoietic stem cell function in mice.[28] To examine the impact of cytokines in BCR-ABL mediated transformation and leukemogenesis, we used SOCS1 as an inhibitor. By modulating cytokine signals *in vitro* and *in vivo* we could demonstrate the importance of cytokine signaling in BCR-ABL induced disease.

## Materials & methods

### Cytokines and antibodies

See S1 File.

### Cell lines

Ba/F3 cells were cultured in RPMI 1640 medium with 10% FCS and 10% conditioned WEHI medium as a source for IL-3. Plat-E cells were grown in DMEM medium with 10% FCS. K562 cells were cultured in RPMI 1640 medium supplemented with 10% FCS, for inhibition of BCR-ABL 2 μM imatinib (Selleckchem, München, Germany) was added to the cells for 16h. Primary murine bone marrow (BM) cells were cultured in DMEM medium supplemented with 10% FCS, stem cell factor [100 ng/ml], IL-3 [20 ng/ml] and IL-6 [20 ng/ml]. Long-term culture of primary human lymphoblastic leukemia cells were grown as described before[29] and when indicated treated with 1 μM imatinib for 16h.

### Mice

Mice were maintained under pathogen-free conditions in the research animal facility of the University Hospital Frankfurt. Animals had free access to food and water, and were housed with a 12-hour light-dark cycle and constant temperature. All animal experiments were performed in accordance with the current ethical standards of the official committee on animal experimentation (written approval by Regierungspräsidium Darmstadt, number V 54–19 c 20/15—F 39/10).

### RNA preparation and RT-PCR

See S1 File.

### Immunoblotting

See S1 File.

### Plasmid construction

PINCO empty vector[30] and PINCO-BCR-ABL (p185) were kind gifts of Dr. Ruthardt (Goethe University, Germany). cDNA of murine CIS and SOCS2 (kind gifts of Dr. Tavernier, Ghent University, Belgium) and SOCS1 were cloned into pENTR1A entry vector (Invitrogen, Regensburg, Germany). SOCS1 and BCR-ABL sequences separated by a T2A peptide sequence were also cloned into pENTR1A vector. pENTR1A vectors encoding SOCS proteins or SOCS1/BCR-ABL were recombined with the PINCO destination vector by a LR clonase reaction. All PINCO constructs co-express EGFP (IRES-CMV-EGFP). For some experiments PAULO-BCR-ABL (p185) was used, which co-expresses truncated human nerve growth factor receptor (NGFR).[31]

### Retroviral transduction

See S1 File.

### Isolation of cells from murine bone marrow

Total BM from C57BL/6 mice was collected by flushing. Lineage negative cells were isolated using a lineage cell depletion kit (Miltenyi Biotec, Bergisch Gladbach, Germany) according to the manufacturer’s instructions. For transplantation of murine BM, Sca-1^+^ cells were used. Briefly, total BM was loaded on a Ficoll gradient. Mononuclear cells were collected, washed and Sca-1^+^ cells were enriched by an anti-Sca-1 MicroBead Kit (Miltenyi Biotec) as recommended by the manufacturer.

### Colony-forming unit assay

See S1 File.

### Transplantation of murine bone marrow

Animals were used with an established local protocol in accordance with the german animal protection law (Tierschutzgesetz § 8, Abs. 1). Intravenous injection and euthanasia of mice occurred under inhalation anesthesia with isoflurane using a vaporizer. Health of animals were monitored daily according to a score sheet approved by the german animal protection law (S2 Table), there were no unexpected deaths. Immediately after the last retroviral transduction, 2.5 x 10^4^ GFP-positive cells were resuspended in 150 μl PBS and injected into the tail vein of sub-lethally irradiated (4,5Gy) female recipient CD45.2^+^ mouse (6–8 weeks old). Peripheral blood was analyzed on day 20 post-transplantation for CD45.1^+^ cells. Mice were sacrificed when a defined assessment score was reached using the mentioned score sheet above. Peripheral blood, BM and spleen cells of moribund mice were analyzed for CD45.1, CD45.2, CD11b, CD19 and Gr-1 expression. At day 200 all remaining mice were sacrificed and analyzed.

### Histology and microscopy

Organs were formalin-fixed and paraffin-embedded, and sections were stained with H&E. Representative images were acquired via an AxioCam camera and Axiovision 4.0 software (Carl Zeiss, Jena, Germany).

### Statistical analysis

Statistical analysis was performed using two-tailed Students’s t-test with GraphPad Prism software package.

## Results

### BCR-ABL induces gene expression of SOCS proteins

**Fig 1:**
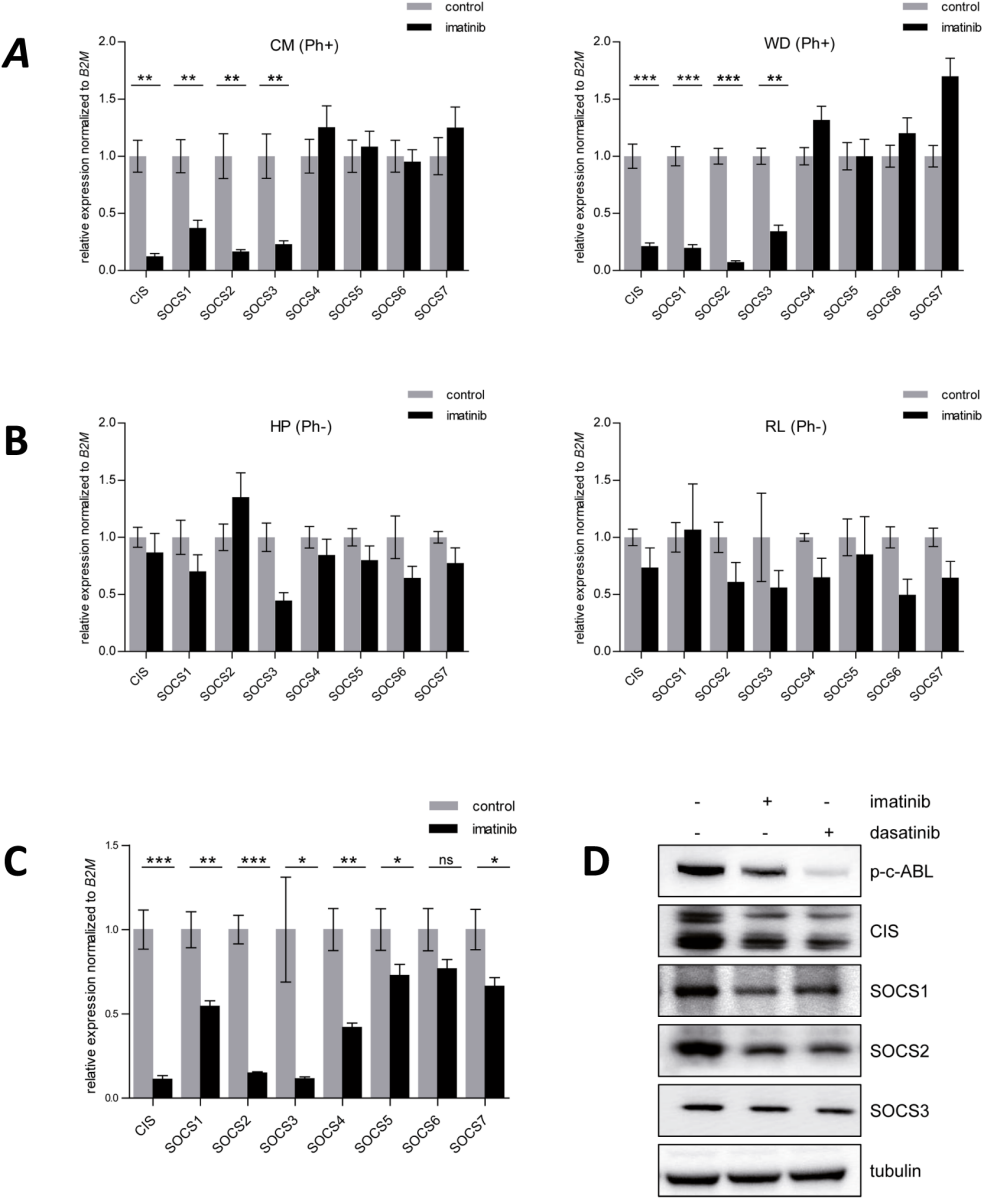
Gene expression levels of SOCS proteins after BCR-ABL kinase inhibition. **A-B.** Philadelphia chromosome positive (Ph+) or negative (Ph-) long term cultures of primary human acute lymphoblastic leukemia cells were treated with 1 μM imatinib for 16h. RNA was extracted and RT-PCR was performed for SOCS family members. Relative gene expression normalized to B2M is shown. The experiment was performed three times, the error bars are indicating the standard deviation. **P ≤ 0.009, ***P ≤ 0.001 **C.** K562 cells were treated with 2 μM imatinib for 16h. RNA was extracted and RT-PCR performed for SOCS family members. Gene expression relative to B2M is depicted. The experiment was performed three times, the error bars are indicating the standard deviation. *P ≤ 0.02, **P ≤ 0.009, ***P ≤ 0.001 **D.** K562 cells were washed with PBS and resuspended in RPMI medium containing 1% FCS. BCR-ABL was inhibited for 16h with 2 μM imatinib or 20 nM dasatinib. Total cell lysates were immunoblotted and probed with indicated antibodies. Tubulin is shown as loading control. Inhibition of BCR-ABL was assessed by its phosphorylation status. The experiment was performed three times.

**Fig 2:**
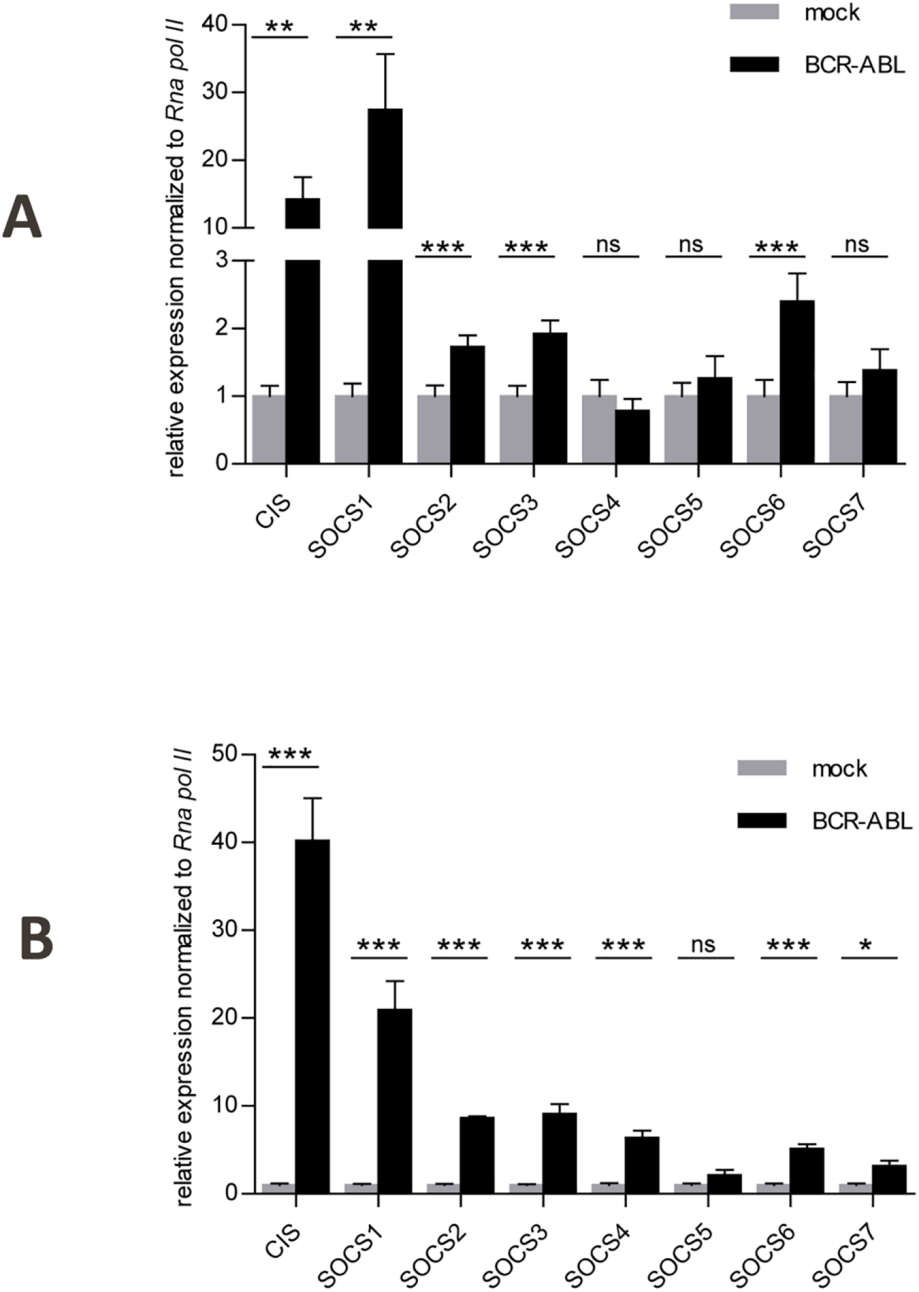
BCR-ABL induces gene expression levels of SOCS proteins. **A.** Ba/F3 cells were transduced with BCR-ABL or an empty vector control. Prior to RNA extraction, cells were starved over night from IL-3. RT-PCR was performed for SOCS family members. Relative gene expression normalized to *RNA pol II* is depicted. The experiment was performed three times, the error bars are indicating the standard deviation. **B.** Sca-1+ cells were enriched from murine BM and transduced with BCR-ABL or an empty vector control. GFP-sorted cells were starved over night from cytokines prior to RNA extraction. Relative gene expression of SOCS family members is shown. Relative gene expression normalized to *RNA pol II* is depicted. The experiment was performed three times, the error bars are indicating the standard deviation. *P ≤ 0.05, **P ≤ 0.009, ***P ≤ 0.001.

Based on our previous findings on SOCS1 in FLT3-ITD mediated leukemia[29] we asked how SOCS family members are regulated by BCR-ABL expressing cells. First BCR-ABL kinase activity was blocked by imatinib in two BCR-ABL positive ALL long-term cultured cells (CM and WD[29]). ABL kinase inhibition by imatinib treatment resulted in down-regulation of gene expression for CIS and SOCS1-SOCS3. SOCS4-SOCS7 displayed either moderate or no regulation (Fig 1A). In contrast, SOCS genes did not respond to imatinib treatment in two BCR-ABL negative long-term cultured cells (HP and RL, Fig 1B). The data suggests that CIS and SOCS1-3 gene expression is dependent on BCR-ABL expression and its kinase activity. Next BCR-ABL positive K562 cells were treated with imatinib and analyzed for gene expression levels of all SOCS family proteins. As in the BCR-ABL positive long-term cultured cells suppression of BCR-ABL led to significant down-regulation of CIS and SOCS1-4 genes as compared to the non-treated controls (Fig 1C). Again SOCS5-7 showed less or no regulation. Importantly the suppressed SOCS gene expression in CIS and SOCS1—SOCS3 led to a decreased SOCS protein expression in imatinib or dasatinib treated K562 cells (Fig 1D and S1 Fig). Next we extended these findings to Ba/F3 cells, transduced with BCR-ABL or an empty vector control. Since cytokines and growth factors themselves can induce SOCS gene expression, we compared the cells after starvation from cytokines, in order to analyze the effects of BCR-ABL and not of e.g. IL-3 or other cytokines on SOCS gene expression. A tremendous up-regulation of CIS and SOCS1 mRNA was observed. A significant effect was also seen in SOCS2, SOCS3 and SOCS6 whereas the remaining SOCS family members were not affected by BCR-ABL (Fig 2A). An analogous experiment in murine primary hematopoietic cells expressing BCR-ABL showed very strong induction of CIS and SOCS1 mRNA (Fig 2B). A moderate, but significant induction of gene expression was seen for the rest of the SOCS proteins except SOCS5. Taken together, CIS, SOCS1 and SOCS2 are induced after BCR-ABL expression suggesting a negative feedback loop to suppress BCR-ABL induced cytokine signaling.

### BCR-ABL confers resistance to SOCS1 expression

**Fig 3:**
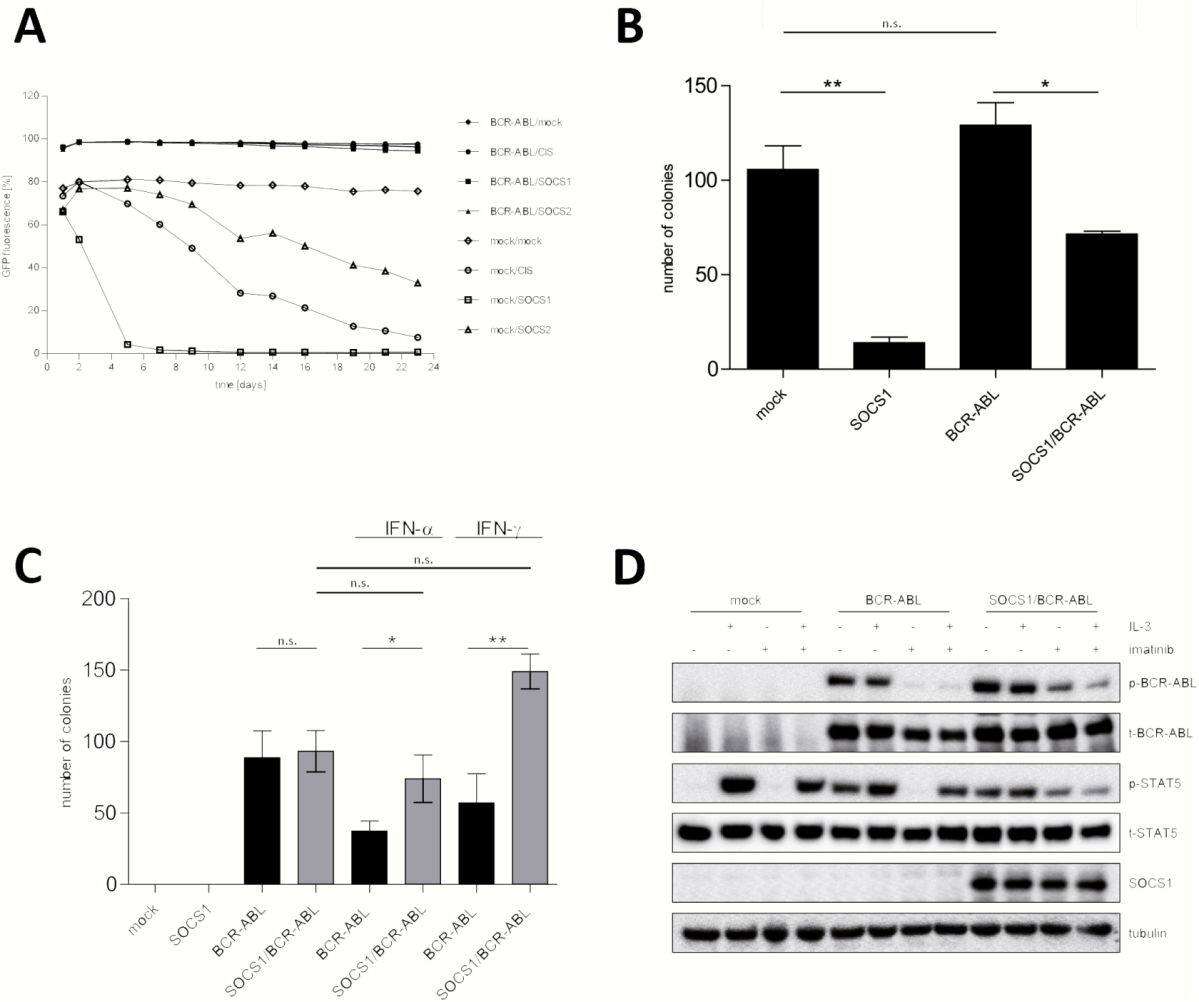
Functional analysis of cells expressing BCR-ABL and/or SOCS1. **A.** IL-3 dependent Ba/F3 cells were transduced with BCR-ABL or a mock control. Cells were sorted via the co-expressed NGFR receptor and then transduced with the indicated SOCS family members or the empty vector control using a vector with IRES-GFP. GFP fluorescence correlating with the expression of SOCS proteins was monitored by flow cytometry. **B.** Lineage negative murine BM cells were transduced with indicated constructs and were sorted for GFP by FACS. 2000 sorted cells per dish were cultured in methylcellulose containing IL-3, IL-6 and SCF. The error bars are indicating the standard deviation. *P ≤ 0.05, **P ≤ 0.006 **C.** 3500 transduced lineage negative murine BM cells (bulk culture) were resuspended in cytokine free methylcellulose per dish. Methylcellulose was supplemented with INF-α [100 ng/ml] or INF-γ [1000 U/ml]. Colony numbers were normalized according to GFP expression measured 48h post transduction. The error bars are indicating the standard deviation. **D.** Ba/F3 cells were incubated in medium containing 0.5% FCS for 6h and treated with 1 μM imatinib in the presence or absence of IL-3. Phosphorylation status of STAT5 was analyzed in total cell lysates. The experiment was performed three times.

To address the function of SOCS proteins and cytokines in BCR-ABL mediated transformation, we expressed different SOCS family proteins in Ba/F3 cells and analyzed their influence on IL-3 dependent proliferation. To select cells, PAULO-BCR-ABL or the empty vector control was introduced into Ba/F3 cells, which were then sorted via the co-expressed nerve growth factor receptor (NGFR). As next these two cell lines were transduced with the different GFP-expressing vectors containing SOCS1, SOCS2, SOCS3, CIS or control. The percentage of GFP-positivity, which correlates with SOCS protein expression, was monitored by flow cytometry (Fig 3A). Starting with 66% GFP-positivity, SOCS1 positive cells died within 5 days (4% GFP on day 5). Also, cells expressing CIS and SOCS2 disappeared from the bulk culture over time, however with slower kinetics. 3 weeks after culture, there were still 8% CIS and 33% SOCS2 positive cells. Mock transduced Ba/F3 cells showed stable GFP signal over time. In contrast, BCR-ABL transduced cells were completely resistant to the action of SOCS proteins, since cells co-expressing BCR-ABL and either CIS, SOCS1 or SOCS2 displayed constant GFP signal. In summary, IL-3 mediated cell proliferation is very sensitive to SOCS1 expression, while BCR-ABL-dependent growth is resistant to SOCS1 co-expression.

### SOCS1 decreases colony numbers under pro-proliferative conditions, while it confers resistance to anti-proliferative cytokines

As functional data from Fig 2 and Fig 3A demonstrated the strongest effects for SOCS1, we focused our efforts on the function of SOCS1 in BCR-ABL mediated transformation and leukemia in the following experiments. For this purpose, we cloned a T2A construct, allowing equimolar expression of BCR-ABL and SOCS1 from the same vector (hereafter abbreviated as SOCS1/BCR-ABL). We transduced the constructs into lineage negative bone marrow (BM) cells and sorted them via the co-expressed GFP. Colony forming capacity of these cells was investigated in methylcellulose containing the pro-proliferative cytokines IL-3, IL-6 and SCF. As expected, also mock-transduced lineage negative cells gave rise to colonies, which was markedly reduced by expression of SOCS1 (Fig 3B). Very low colony numbers detected for SOCS1 single transduced cells are due to inhibition of IL-3 and IL-6 signaling. Importantly, in BCR-ABL positive cells, co-expression of SOCS1 decreased colony growth, although the reduction was much less pronounced than with cells expressing SOCS1 only suggesting a partial resistance. When the colony assay was performed in cytokine free methylcellulose the empty control or SOCS1 expressing cells did not grow (Fig 3C). BCR-ABL can cause proliferation in a growth factor independent manner, thus, colony formation was very similar in BCR-ABL and SOCS1/BCR-ABL transduced cells. However, when methylcellulose was supplemented with the anti-proliferative cytokines IFN-α or IFN-γ, SOCS1/BCR-ABL expressing cells formed significantly more colonies than the BCR-ABL positive counterparts. This phenomenon is due to the fact that SOCS1 in SOCS1/BCR-ABL cells inhibits the action of these interferon, thereby eliminating their anti-proliferative effects. In contrast, in cells expressing BCR-ABL alone interferon negatively influence cell proliferation and thus decrease colony numbers. In summary, SOCS1 is lowering colony forming capacity of primary murine BM cells under pro-proliferative conditions, while it protects cells from the effects of anti-proliferative cytokines indicating the potential tumor suppressive but also oncogenic function of SOCS1.

### SOCS1 reduces IL-3 induced phosphorylation of STAT5 in SOCS1/BCR-ABL cells

BCR-ABL exerts its function by converting the tightly controlled kinase activity of c-ABL into a constitutive active form. Thus, we investigated if the co-expression of SOCS1 changes central signaling events downstream of BCR-ABL in Ba/F3 cells. As STAT5 plays a key role in the initiation and maintenance of BCR-ABL mediated leukemia[32,33], we performed a more detailed analysis in this context. In Ba/F3 cells, IL-3 and BCR-ABL are very strong inducers of STAT5 phosphorylation, and BCR-ABL dependent phosphorylation is inhibited by imatinib (Fig 3D and S1 Fig). Withdrawal of IL-3 slightly reduced the activation of STAT5 in cells expressing BCR-ABL or SOCS1/BCR-ABL. When BCR-ABL activity was blocked by imatinib in the presence of IL-3, phosphorylation of STAT5 was sustained due to IL-3 stimulation. In contrast, in imatinib treated SOCS1/BCR-ABL positive cells, STAT5 phosphorylation was markedly reduced in the presence of IL-3, although inhibition of BCR-ABL was not as efficient as in BCR-ABL only transduced cells. Thus, SOCS1 over-expression was able to inhibit IL-3 mediated phosphorylation of STAT5.

### BCR-ABL driven leukemia is sensitive to the cytokine microenvironment

**Fig 4:**
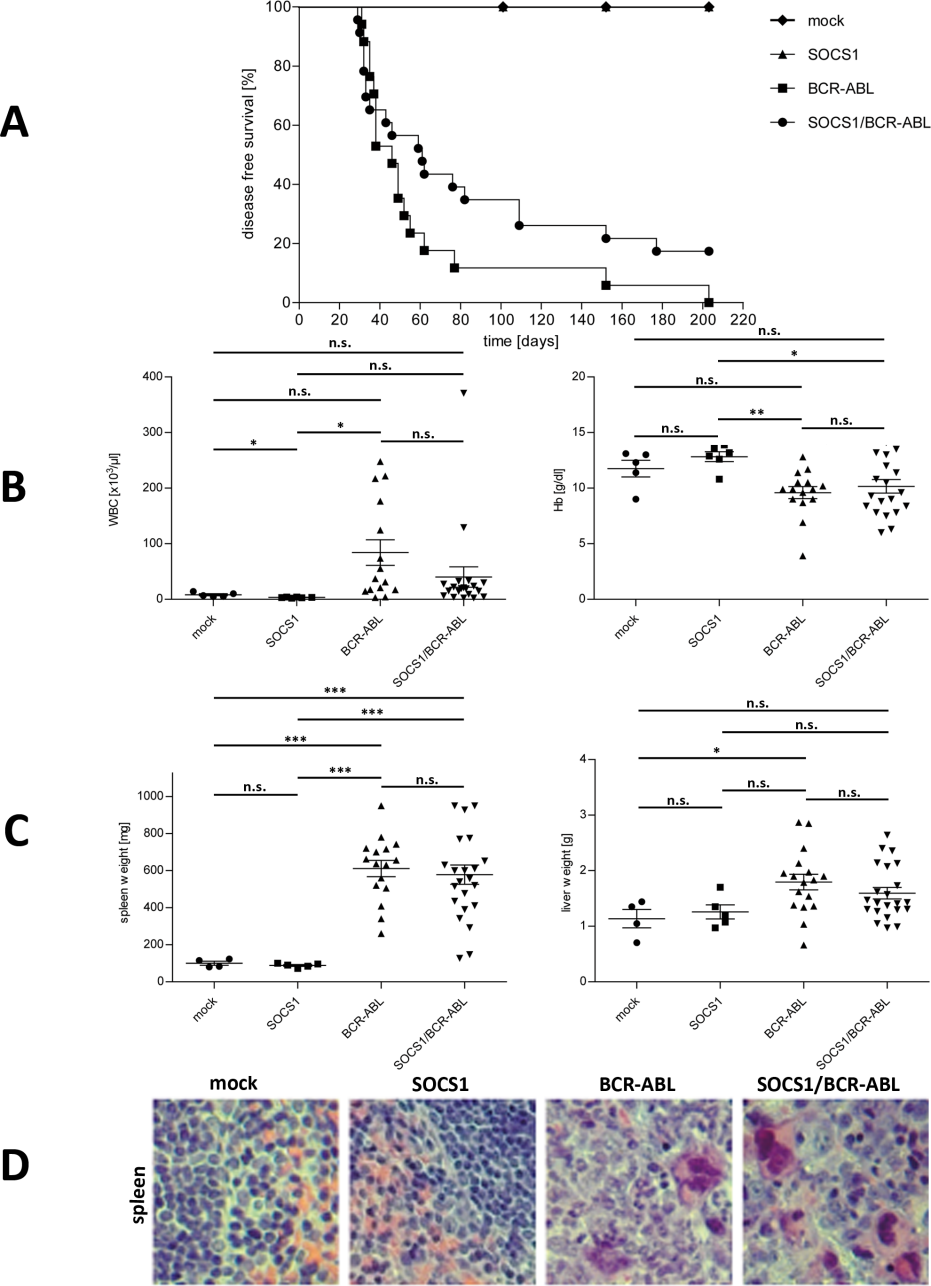
Mouse transplantation model. Transduction efficiency of Sca-1^+^ cells was normalized according to GFP expression and 2.5 x 10^4^ GFP-positive cells were transplanted into the tail vein of sub-lethally irradiated recipient mice. Moribund mice were sacrificed and analyzed. All data shown are from time of death. **A.** Disease free survival of transplanted animals is plotted in Kaplan-Meier curves. **B.** White blood counts and hemoglobin content in the peripheral blood. **C.** Spleen and liver weights of analyzed mice. **D.** Spleen samples were stained with H&E. Representative images are shown.

To study the impact of the cytokine environment in BCR-ABL induced disease, we co-expressed SOCS1 and BCR-ABL *in vivo* in a mouse BM transplantation model. Because of possible silencing of the CMV promoter in vivo driving GFP expression [34,35], we made use of the congenic CD45 allele to track the donor cells in the recipient mice. 20 days post transplantation peripheral blood was analyzed for the presence of CD45.1^+^ donor cells. Animals negative for donor cells at this time point were excluded from the experiment. Moribund mice were sacrificed and subjected to detailed analysis. Kaplan-Meier analysis of disease free survival revealed surprising results. As expected, mock and SOCS1 transplanted animals did not develop leukemia. They were sacrificed either for control purposes (censored) or to terminate the experiment (beyond day 200). BCR-ABL and SOCS1/BCR-ABL transplanted mice were sacrificed at onset of severe signs of disease (Fig 4A). Interestingly, in both groups mice died initially with similar kinetics. However, at day 50 survival curves separated with animals in the SOCS1/BCR-ABL group surviving longer. At day 100, the survival in the BCR-ABL and SOCS1/BCR-ABL was 12% and 35%, respectively. Notably, four mice (20%) in the SOCS1/BCR-ABL group did not develop any disease although two of them had enlarged spleens when the experiment was terminated. Interestingly, two of these four mice—being positive for CD45.1 after transplantation—lost donor cells during time and did not exhibit any signs of leukemic development. As a hallmark of leukemia, diseased mice exhibited elevated white blood counts (WBC), which was accompanied by decreased hemoglobin (Fig 4B). Spleens of sacrificed animals were characterized by splenomegaly and in some cases weighed up to 1 g (Fig 4C left panel). Distribution of spleen weights in BCR-ABL and SOCS1/BCR-ABL groups were comparable and did not show any significant differences. An enlargement of the livers was also detected, however, not as strikingly as for the spleen (Fig 4C right panel). Macroscopic analysis of peripheral blood, BM and spleen cells revealed marked differences between the control mice on the one hand, and BCR-ABL or SOCS1/BCR-ABL groups on the other. Smears of the peripheral blood of mice from the BCR-ABL or SOCS1/BCR-ABL groups displayed high counts of differentiated myeloid cells, suggestive of MPD (data not shown). Increased numbers of these cells were also detected in spleens. Histological examination of spleen tissues confirmed destruction of normal tissue architecture with mature granulocytes in mice from the BCR-ABL or SOCS1/BCR-ABL groups (Fig 4D). All mice that developed disease suffered from a MPD, except two animals (one in each group) that died from B-ALL (acute lymphoblastic leukemia) (Table 1). Taken together, the co-expression of SOCS1 and BCR-ABL prolonged the disease latency and prevented disease in a subset of mice, most likely by shutoff of predominantly pro-proliferative cytokine signals in the hematopoietic microenvironment.

**Table 1:** BM and splenocyte analysis of transplanted mice at the time of death.

	No. of mice	Phenotype	BM			Spleen		
			%CD45.1	%CD19	%CD11b/Gr-1	%CD45.1	%CD19	%CD11b/Gr-1
Control	7	none	2.3	21.2	14.3	11.4	27	0.6
			(1.1–3.3)	(1.2–48.6)	(3.8–23.4)	(0–24.3)	(0–49.7)	(0–1.2)
SOCS1	8	none	7.5	15.3	23.4	14	34.3	0.5
			(0–25.4)	(0–50.8)	(2–63.9)	(0–51.6)	(0–58.2)	(0–1.2)
BCR-ABL	17	16 MPD	65.9	11.9	77	71.3	12	63.4
			(6.9–93.3)	(0–88.5)	(23.8–92.4)	(6.9–93.3)	(0–89.9)	(11.1–92.3)
		1 B-ALL	59.1	99	0.4	60.6	96.9	0.1
SOCS1/	21	16 MPD	52	5.5	69.1	56.8	18	56.3
BCR-ABL			(15.9–89.2)	(0–19)	(33.3–97.7)	(14–86.3)	(1.1–41.1)	(4.5–86)
		1 B-ALL	87.6	93.1	0.1	73.2	91	2
		4 no disease	3.9	13.4	21.5	1.4	22.7	9.4
			(1.7–7.8)	(11.2–17.4)	(7.8–35)	(0.8–2.2)	(15.6–30.7)	(2.4–18.5)

## Discussion

Although application of TKIs such as imatinib turned CML from a life-threatening condition to a treatable disease, eradication of leukemia initiating stem cells is still an unsolved problem. One of the factors causing TKI resistance is the cytokine environment that supports growth and survival of CML stem cells irrespective of BCR-ABL activity.[14] In line with this, disruption of the cytokine support in BCR-ABL induced lymphoblastic leukemia enhances sensitivity to imatinib.[36] To further elucidate the function of cytokines in BCR-ABL mediated transformation and leukemogenesis we used SOCS1 as a tool to modulate cytokine signals.

As a physiological phenomenon BCR-ABL transformation unequivocally leads to up-regulation of cytokine signal inhibition most likely in response to STAT5 phosphorylation. When endogenous BCR-ABL was inhibited by tyrosine kinase inhibitors in BCR-ABL positive ALL long-term cultured cells and in K562 cells, CIS and SOCS1-3 showed comparable down-regulation of gene and protein expression (Fig 1). SOCS4-7 exhibited less or no regulation. Reciprocally, ectopic expression of BCR-ABL in primary cells and cell lines resulted in highest induction of CIS and SOCS1 gene expression (Fig 2). These two types of responses might be reflected by the presence of two “sub-families” within the SOCS proteins. CIS and SOCS1-3 have shorter N-terminal regions compared to SOCS4-7. The first group is believed to be more involved in regulation of cytokine receptor signaling via the JAK-STAT signaling pathway while SOCS4-7 are suggested to function primarily in growth factor receptor signaling.[18] Since SOCS1 exerted a very potent inhibition of the IL-3 dependent proliferation of Ba/F3 cells compared to CIS and SOCS2 (Fig 3A), we focused our efforts on the role of SOCS1 in BCR-ABL induced transformation and leukemia. In colony forming unit assays with murine BM cells SOCS1 reduced colony numbers under pro-proliferative conditions when co-expressed with BCR-ABL. Under anti-proliferative conditions, co-expression of SOCS1 conferred a BCR-ABL dependent growth advantage since the cells were not affected by IFN-α or IFN-γ in contrast to the BCR-ABL expressing cells (Fig 3B and 3C). IFN-α administration was a major type of therapy in the treatment of CML before imatinib was available. In a group of Philadelphia positive chronic phase CML patients poor response to IFN-α was monitored[37]. Strikingly, these patients displayed constitutive expression of SOCS1 which was the only factor predicting the cytogenetic response to IFN-α.[37] This observation is in accordance with the results of our CFU assay, confirming the resistance to IFN-α in cells co-expressing SOCS-1 and BCR-ABL. A more profound understanding for the cytokine environment of leukemic cells and for the function of SOCS1 as an oncogene or as a tumor suppressor in BCR-ABL mediated transformation might be required to completely eradicate leukemia-initiating cells.

BCR-ABL activates different signal cascades in the cell.[38] Under steady state conditions, the co-expression of SOCS1 did not alter downstream signaling of BCR-ABL. However, when BCR-ABL was inhibited by ABL kinase inhibitors, SOCS1 reduced IL-3 driven phosphorylation of STAT-5 in Ba/F3 cells. In BCR-ABL induced leukemia STAT5 is indispensable. Strong evidence suggests that BCR-ABL directly phosphorylates STAT5.[39] Thus, a cell that is transformed by BCR-ABL does not need any additional signaling to activate STAT5. Nevertheless, it is imaginable that there is a dose dependent response. Cytokines activating STAT5 additionally such as IL-2, IL-3, IL-7, GM-CSF and many others could enhance the effect of BCR-ABL and promote leukemia induction and maintenance. This additional signaling might be inhibited by SOCS1 explaining a prolonged latency in the mouse model.

When transplanted into mice, BCR-ABL and SOCS1/BCR-ABL induced leukemia with massive infiltration of hematopoietic tissues with differentiated myeloid cells. Disease onset was fast in both groups with mainly MPD phenotype. In humans, BCR-ABLp185 is associated with a B-ALL phenotype while BCR-ABLp210 is causing CML.[7] However, in mouse models this is very much dependent on the cell type that is used for transduction and transplantation as well as the cytokines included during the *in vitro* culture.[40] Disease kinetics in both groups was initially very similar, however, the co-expression of SOCS1 and BCR-ABL prolonged disease formation from day 50 onwards. Interestingly, about 20% of the mice in this group did not develop any disease (Fig 4A). In the BM of these mice only ~4% CD45.1^+^ cells were present 200 days after transplantation. In contrast, when mice in the same group died from MPD an average of 52% of CD45.1^+^were detected in the BM. Failure in homing and/or repopulation can be excluded since 20 days after transplantation these mice were positive for CD45.1 cells in their peripheral blood (23–68%). Two of these mice even showed splenomegaly with no signs of leukemic disease. In these animals the presence of SOCS1 seemed to entirely protect from BCR-ABL mediated leukemia. Zhang and Ren[9] showed increased gene transcripts and elevated serum levels of IL-3 and GM-CSF in BCR-ABL transplanted mice compared to the vector controls. This indicates that the BCR-ABL transformed cells have developed strategies to enhance cytokine production supporting its survival and proliferation. Hence, we assume that especially in the onset of myeloproliferative diseases pro-proliferative conditions dominate over anti-proliferative. SOCS1 over-expression reduces cytokine signals *in vivo* and can thereby decelerate and even prevent BCR-ABL mediated MPD.

The outcome of the *in vivo* transplantation model markedly differs from the results we obtained with SOCS1-FLT3-ITD. When co-expressed with FLT3-ITD, SOCS1 shortened disease latency.[41] Still, a direct comparison of these mouse transplantation models is difficult due to completely different experimental setups. In both studies different vectors, source of cells and mice strains were employed, strongly influencing the experimental outcome. On the other hand, activity of SOCS1 is dependent on the oncogene and a general mode of action is unpredictable. When Rottapel *et al*.[42] transplanted Ba/F3 cells transduced with Tel-Jak2 or BCR-ABL co-expressing SOCS1 into nude mice, SOCS1 abrogated tumorigenicity of Tel-Jak2 while SOCS1/BCR-ABL transduced cells still induced tumors. However, formation of metastasis was blocked by the presence of SOCS1 in the SOCS1/BCR-ABL group supporting a tumor suppressive role for SOCS1. Qiu *et al*.[43] presented a BCR-ABL-dependent tyrosine phosphorylation of SOCS-1 and SOCS-3 as an escape mechanism to overcome inhibition by those proteins. When the respective phosphorylation sites were mutated in SOCS-1 or SOCS-3, BCR-ABL induced tumor formation was completely blocked in nude mice due to “activation” of SOCS-1 or SOCS-3. Similarly, in transformed pre-B cells v-ABL signaling leads to SOCS1 phosphorylation thereby altering SOCS1-dependent proteasomal degradation of JAKs.[44] This might explain why we do not see a complete blockage but rather a suppression of leukemia development in our system. Moreover, we showed BCR-ABL dependent induction of SOCS1 expression. Thus, in the mouse transplantation model we compare BCR-ABL transduced cells with low SOCS1 expression to cells over-expressing BCR-ABL and SOCS-1, which probably weakens the difference in disease progression between these groups. The cleaner experiment would be to transduce BM from SOCS1 knock-out mice with BCR-ABL, which is unfortunately not possible due to postnatal lethality of the SOCS1 knock-out. Tumor suppressing activity of SOCS1 is also supported by the high frequency of SOCS1 gene silencing by DNA hypermethylation in different cancer cell lines and in human malignant diseases such as multiple myeloma[45], acute myeloid leukemia[46,47], hepatocellular carcinoma[48], ovarian and breast carcinomas.[25] In CML, however, results of epigenetic analyses of the SOCS1 gene are conflicting. Although the same region was analyzed, Hatirnaz *et al*.[49] could not detect any methylation of the SOCS1 promoter in CML patients, while others demonstrated SOCS1 gene silencing by promoter hypermethylation.[50]

Taken together our findings give an explanation why such diverse und contradicting roles are described for SOCS proteins in cancer. As we have shown, SOCS1 can act as both a tumor suppressor and an oncogene in BCR-ABL mediated transformation. In pro-proliferative cytokine environment—and we assume that particular situation at the onset of myeloproliferative diseases—SOCS1 suppresses the predominantly pro-proliferative cytokines and thus prolongs or even prevents BCR-ABL mediated transformation. If however an anti-proliferative cytokine environment is present—which might well be the case at other stages of myeloproliferative disease or other diseases in general—SOCS1 exerts a very different function. It inhibits the anti-proliferative cytokine signaling and thereby supports malignant transformation. Future works on SOCS1 and the other SOCS family member need to consider the cytokine environment *in vivo* and *in vitro* when biological readouts of SOCS function are made. In the long term new strategies for the therapy of BCR-ABL should consider the cytokine environment as a therapeutic target and SOCS1 as tool to modulate this.

## Supporting information

S1 File Containing supplemental information. (DOCX)

S1 Table Primer sequences. (DOCX)

S2 Table Score sheet for assessment of mice in animal experiments. (DOCX)

S1 Fig Gene expression levels of SOCS proteins after BCR-ABL kinase inhibition. Densitometric analyses were performed for one representative western blot. The densitometric values were normalized to tubulin and then adjusted proportionate to the control that was set as 1.00. **A.** K562 cells were treated for 16h with 2 μM imatinib or 20nM dasatinib. **B.** Ba/F3 cells were incubated in medium containing 0.5% FCS for 6h and treated with 1 μM imatinib in the presence or absence of IL-3.
(DOCX)
