# Supplementary material for: SOCS1 function in BCR-ABL mediated myeloproliferative disease is dependent on the cytokine environment
Source: PLoS One. 2017 Jul 28;12(7):e0180401. doi: 10.1371/journal.pone.0180401 (PMC5533340; doi:10.1371/journal.pone.0180401)
Supplement: S1 File — (DOCX) [file pone.0180401.s001.docx]

**S1 File - Supplemental information**

**Cytokines and antibodies**

All cytokines were purchased from PeproTech (Hamburg, Germany) except IFN-α which was from PBL Interferon Source (New Jersey, USA). Anti-phospho-STAT5 (Tyr 694), anti-phospho-c-ABL (Tyr 245), anti-CIS, anti-SOCS2 and anti-SOCS3 antibodies were obtained from Cell Signaling Technology (Frankfurt am Main, Germany). Anti-STAT5 were purchased from Santa Cruz Biotechnology (Santa Cruz, USA). Anti-c-ABL was obtained from Calbiochem (Darmstadt, Germany). Anti-SOCS1 was purchased from Millipore (Darmstadt, Germany) and Cell Signaling Technologyand anti-γ-tubulin from Sigma-Aldrich (Steinheim, Germany). NGFR antibody (PE, clone C40/1457), CD45.1 antibody (PE, clone A20), CD45.2 antibody (V450, clone 104), CD11b antibody (PE, clone M1/70), CD19 antibody (APC-H7, clone 1D3) and Gr-1 antibody (APC, clone RB6-8C5) for FACS analysis were purchased from BD Biosciences (Heidelberg, Germany).

**RNA preparation and RT-PCR**

Total RNA was isolated using RNA-Bee (AMS Biotechnology, Abingdon, UK) according to the manufacturer’s instructions and transcribed into cDNA using RevertAid H minus first strand cDNA synthesis kit (Thermo Scientific, Darmstadt, Germany). Relative gene expression levels were calculated using standard curves generated by serial dilutions of the analyzed cDNA and displayed as relative expression to beta-2-microglobulin (*B2M*). Sequences of primers and probes are provided in S1 Table.

**Immunoblotting**

Cells were either lysed by sonication in buffer containing 100 mM Tris, 10% SDS, 150 mM NaCl, 10 mM EDTA, protease inhibitor mix (Roche, Mannheim, Germany) and 1 mM sodium orthovanadate or lysed in RIPA buffer containing protease inhibitor mix and 1 mM sodium orthovanadate on ice for 30 min. Protein concentration was determined with the DC protein assay kit (Bio-Rad, München, Germany) and equal amount of protein was separated on Bis-Tris gels. For phosphoprotein analysis, cells were intensively washed with PBS and starved in culture medium containing 0.5% FCS for 6h. When indicated, medium was supplemented with 1or 2 µM imatinib or 20 nM dasatinib (Selleckchem).

**Retroviral transduction**

Ecotropic retroviral particles were produced in Plat-E cells by transfection of plasmid DNA using Turbofect (Fermentas, Darmstadt, Germany) according to the manufacturer’s instructions. Supernatants were collected 48 and 72h post transfection. For retroviral transduction RetroNectin (Takara, Saint Germain En Laye, France) precoated plates were used. At first 20ng/ml RetroNectin was added on 6 well culture plates (Sigma-Aldrich) for 2h at room temperature, bovine serum albumin (BSA) was added for 30min and then washed with HBSS buffer. The retroviral containing supernatant was centrifuged on the plate for 45min at 1100 x g, this step was repeated for a second time. Primary cells were resuspended in DMEM medium supplemented with 10% FCS, stem cell factor [100 ng/ml], IL-3 [20 ng/ml] and IL-6 [20 ng/ml] and polybrene 5µg/ml. Ba/F3 cells were resuspended in RPMI 1640 medium with 10% FCS and 10% conditioned WEHI medium as a source for IL-3 After recovery for at least four hours, transduction was repeated. On day 3, transduction was performed again for two rounds, GFP expression was determined and cells were immediately transplanted into recipient mice.

**Colony-forming unit assay**

Transduced lineage negative cells were sorted for GFP on a FACSAria cell sorter (BD Biosciences). 2000 cells per dish were resuspended in methylcellulose containing cytokines (M3534, Stemcell Technologies, Köln, Germany). For cytokine free experiments, lineage negative cells were transduced and bulk cultures were used 48h post transduction. 3500 lineage negative cells were cultured in cytokine free methylcellulose (M3234, Stemcell Technologies). When indicated methylcellulose was supplemented with INF-α [100 ng/ml] or INF-γ [1000 U/ml]. For each condition triplicates were performed. Colony numbers were normalized according to GFP expression measured 48h post transduction.
