# Supplementary material for: SOCS1 function in BCR-ABL mediated myeloproliferative disease is dependent on the cytokine environment
Source: PLoS One. 2017 Jul 28;12(7):e0180401. doi: 10.1371/journal.pone.0180401 (PMC5533340; doi:10.1371/journal.pone.0180401)
Supplement: S1 Table — (DOCX) [file pone.0180401.s002.docx]

**S1 Table - Primer sequences**

| murine | forward primer [5‘-3‘] | reverse primer [5‘-3‘] | probe [5‘-3‘] |
| --- | --- | --- | --- |
| CIS | CGGGAATCTGGGTGGTACTG | ACCCTCCGGCATCTTCTGTA | TCTATTACAGCCAGCGAGGCCCGG |
| SOCS1 | CCGTGGGTCGCGAGAAC | AAGGAACTCAGGTAGTCACGGAGTA | TGGCGCGCATCCCTCTTAACCC |
| SOCS2 | TCCAGATGTGCAAGGATAAACG | GGTTTGGTCAGGTACAGGTGAAC | CAGGCCCAGAAGCCCCACGG |
| SOCS3 | CCACCCTCCAGCATCTTTGT | CAGGCAGCTGGGTCACTTTC | ACTGTCAACGGCCACCTGGACTCCT |
| SOCS4 | TGTTCTTTGAGCCGCTCTTGT | TGCAAATATGCTGCAAGGAAA | CACTCCCTTAATCCGGACGTTCCCC |
| SOCS5 | TTTCCAGGCGGAACCAAA | TGTCTTTCTCGATGCTGATTTCA | CTGTGCCGCAGAGATCCCTCAAGTG |
| SOCS6 | AACCCAATCCAAAGGAACTTCA | CGAACACTTTCGGCCATGT | TGGCCTCTCGGGCCCAGACTT |
| SOCS7 | TTGCAGTCTTTCCCCCTACCT | ACGCTCTGATGGGAGCAATT | ACCCCACGCCCCAGATGCGT |
| B2M | CCTTGAGGCTATCCAGCGT | CCTGCTCAGATACATCAAACATG | CCTCCATGATGCTGCTTACATGTCTC |
| POL-II | TGTTGGTGTATTGCCTGGCT | GGACATGGAGCACCTTGGAA | Mm00839502_m1 |

| human | forward primer [5‘-3‘] | reverse primer [5‘-3‘] | probe [5‘-3‘] |
| --- | --- | --- | --- |
| CIS | CAGACAGAGAGTGAGCCAAAGGT | ACCCCAATACCAGCCAGATTC | TCTGCTGTGCATAGCCAAGACCTTCTCC |
| SOCS1 | CCAGCGGAACTGCTTTTTC | GCTGCCATCCAGGTGAAAG | CTTAGCGTGAAGATGGCCTCGGGA |
| SOCS2 | GAACGGCACTGTTCACCTTTATC | GCCTACAGAGATGCTGCAGAGA | ACCAAACCGCTCTACACGTCAGCACC |
| SOCS3 | CCAGCCTGCGCCTCAA | CTTGCGCACTGCGTTCAC | CCTTCAGCTCCAAGAGCGAGTACCAGC |
| SOCS4 | GAAGATAGTGATATGGATTCCGATGA | TGTTTCCAACTGCAGGATTTCA | TTCCAGAAAAAGAAACAAACCCAAATGGGA |
| SOCS5 | CAGGGACTCTGCGCAAGAG | CGGGCATGCAGGGATCT | ACTACCTCTTCTCTGTGAGCTTCCGCCG |
| SOCS6 | TCCACGACCTCCAGTCTGAGA | ATTATGAGAAGCCGAGCTCTTCA | CGTGCCAGGAGCAAGCCAATTCA |
| SOCS7 | CCCGATTCAGCAATGTCAAA | AGAGGTTTAGGCAGTGGGAGATC | AGCACCTTTGCAGATTCCGGATACGA |
