# Supplementary material for: SOCS1 function in BCR-ABL mediated myeloproliferative disease is dependent on the cytokine environment
Source: PLoS One. 2017 Jul 28;12(7):e0180401. doi: 10.1371/journal.pone.0180401 (PMC5533340; doi:10.1371/journal.pone.0180401)
Supplement: S2 Table — (DOCX) [file pone.0180401.s003.docx]

**S2 Table - Score sheet for assessment of mice in animal experiments**

| **Behavior:** |  |
| --- | --- |
| Normal: attentive, social contacts | N |
| Reduced reaction | A |
| Crouching pose, isolation from group, slow movement, lethargic | B |
| Apathetic, impaired movement, isolation from group, propped up head | C |
|  |  |
| **Physical appearance:** |  |
| Normal: no signs of disease | N |
| Missing Grooming | A |
| Partially shaggy fur particularly in the throat area, nasal discharge | B |
| Signs of a water deficiency In the body (exsiccosis) | B |
| Clouded eyes | B |
| Completely shaggy fur | C |
|  |  |
| **Breathing:** |  |
| Normal breathing | N |
| Slightly more visible respiration | A |
| Markedly increased respiration | B |
| Mucosal cyanosis, fighting for air | C |
|  |  |
| **Body weight changes:** |  |
| Normal corresponding to the weight of the strain and the same age | N |
| Weight deficit <10% | A |
| Weight deficit 10-20% | B |
| Weight deficit >20% | C |
|  |  |
| **Local changes:** | A |
| Redness (Rubor) | A |
| Pain (Dolor) | A |
| Heat (Calor) | A |
| Swelling (Tumor) | A |
| Loss of function (Functio laesa) | B |
| Formation of pus | B |
| Licking, scratching, scrubbing | C |
| Automutilation |  |
|  |  |
| **Signs of pain or suffering:** |  |
| Shaggy unkempt fur | B |
| Gait disorder (ataxia) | B |
| Stretching of lower limbs | C |
| Vocalizations | C |
| Isolation from group | B |
|  |  |
| **Neurological disorders:** |  |
| Trembling | B |
| Fluctuating movements | B |
| Cramps | C |
| Circulation movements | C |
| Paresis | C |
| Paralysis | C |

**Consequences:**

1 x A: continue daily examination

>2 x A: presentation to veterinarian or project manager

B: presentation to veterinarian or project manager and analgesic medication

2 x B or 1 x C: end of trial, euthanasia
