# Supplementary figures and images for: SOCS1 function in BCR-ABL mediated myeloproliferative disease is dependent on the cytokine environment
Source: PLoS One. 2017 Jul 28;12(7):e0180401. doi: 10.1371/journal.pone.0180401 (PMC5533340; doi:10.1371/journal.pone.0180401)

**S1 Fig - Supplemental Figure 1**

**
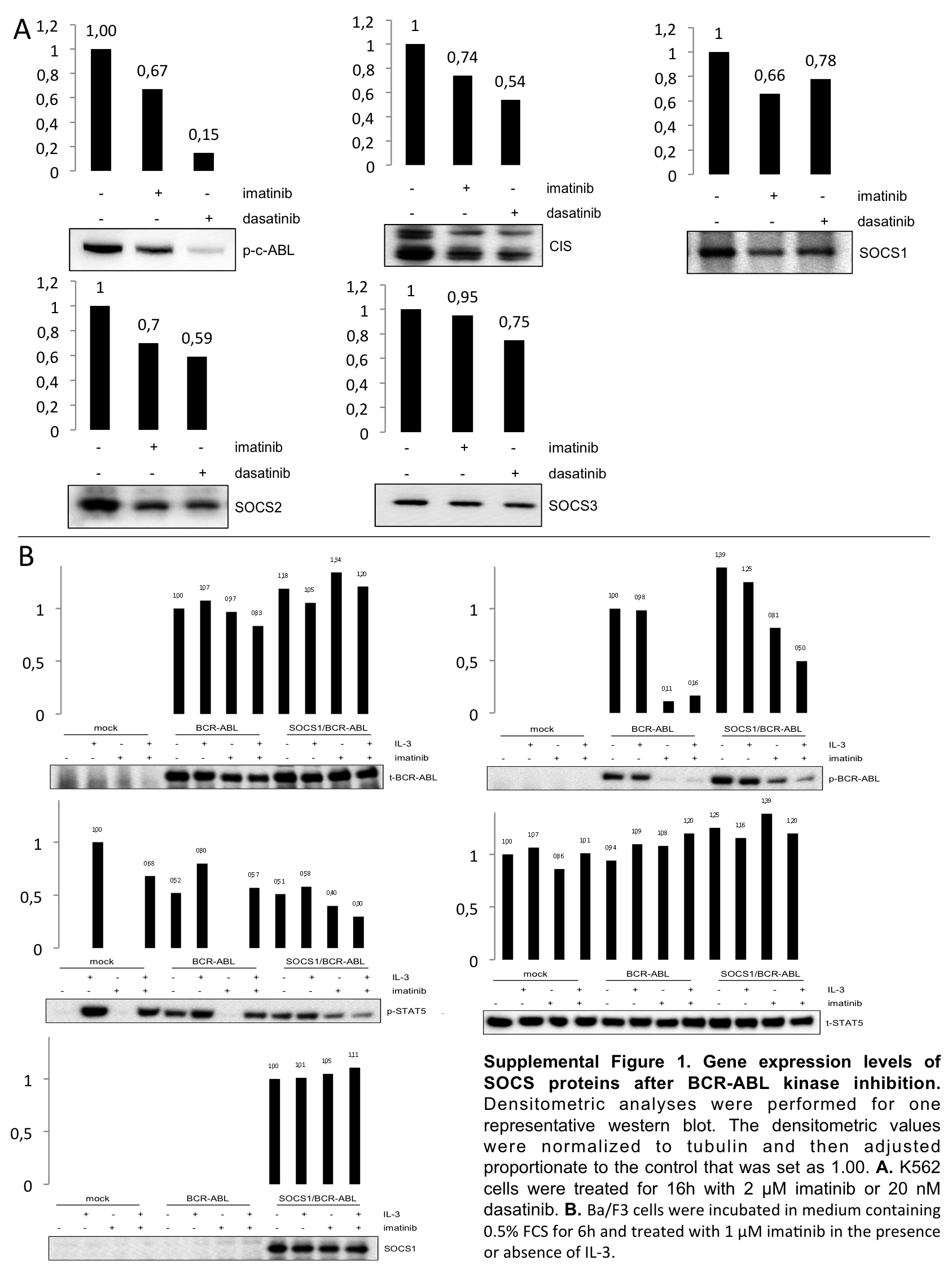
**

Supplement: S1 Fig — Densitometric analyses were performed for one representative western blot. The densitometric values were normalized to tubulin and then adjusted proportionate to the control that was set as 1.00. A. K562 cells were treated for 16h with 2 μM imatinib or 20nM dasatinib. B. Ba/F3 cells were incubated in medium containing 0.5% FCS for 6h and treated with 1 μM imatinib in the presence or absence of IL-3. (DOCX) [file pone.0180401.s004.docx]
